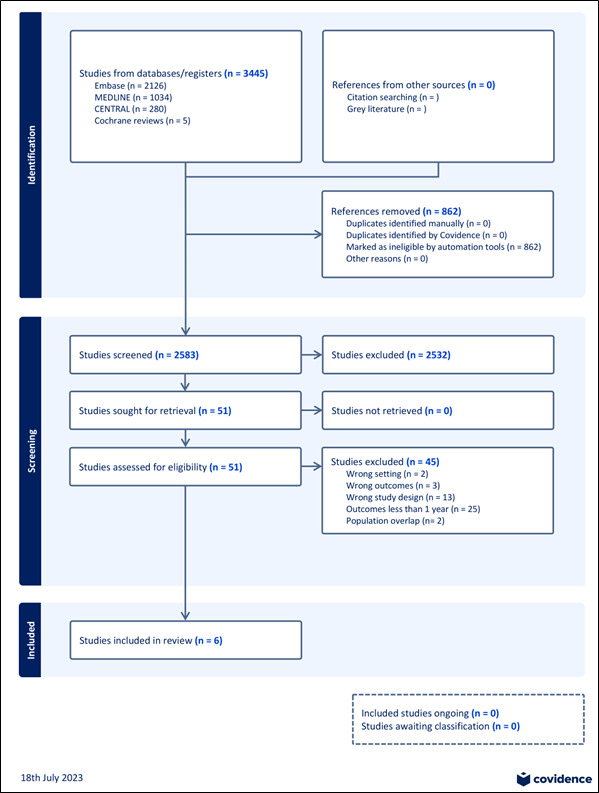# Re-exploration for bleeding and long-term survival after adult cardiac surgery: a meta-analysis of reconstructed time-to-event data

**DOI:** 10.1097/JS9.0000000000001765

**Published:** 2024-06-07

**Authors:** Giovanni Jr Soletti, Gianmarco Cancelli, Michele Dell’Aquila, Tulio Caldonazo, Lamia Harik, Camilla Rossi, Panagiotis Tasoudis, Jordan Leith, Kevin R. An, Arnaldo Dimagli, Michelle Demetres, Mario Gaudino

**Affiliations:** a Department of Cardiothoracic Surgery at New York Presbyterian, Weill Cornell Medicine, New York; b Division of Cardiothoracic Surgery, University of North Carolina, Chapel Hill, USA; c Department of Cardiothoracic Surgery, Friedrich-Schiller-University Jena, Germany; d Samuel J. Wood Library & CV Starr Biomedical Information Center, Weill Cornell Medicine, New York, NY

**Keywords:** cardiac surgery, mortality, postoperative bleeding, re-exploration

## Abstract

**Background:**

Postoperative bleeding requiring re-exploration is a serious complication that occurs in 2.8–4.6% of patients undergoing cardiac surgery. Re-exploration has previously been associated with a higher risk of short-term mortality. However, a comprehensive analysis of long-term outcomes after re-exploration for bleeding has not been published.

**Materials and methods:**

The authors performed a systematic, three databases search to identify studies reporting long-term outcomes in patients who required re-exploration for bleeding after cardiac surgery compared to patients who did not, with at least 1-year of follow-up. Long-term survival was the primary outcome. Secondary outcomes were operative mortality, myocardial infarction, stroke, renal and respiratory complications, and hospital length of stay. Random-effects models was used. Individual patient survival data was extracted from available survival curves and reconstructed using restricted mean survival time.

**Results:**

Six studies totaling 135 456 patients were included. The average follow-up was 5.5 years. In the individual patient data, patients who required re-exploration had a significantly higher risk of death compared with patients who did not [hazard ratio (HR): 1.21; 95% CI: 1.14–1.27; *P*<0.001], which was confirmed by the study-level survival analysis (HR: 1.32; 95% CI: 1.12–1.56; *P*<0.01). Re-exploration was also associated with a higher risk of operative mortality [odds ratio (OR): 5.25, 95% CI: 4.74–5.82, *P*<0.0001], stroke (OR: 2.05, 95% CI: 1.72–2.43, *P*<0.0001), renal (OR: 4.13, 95% CI: 3.43–4.39 *P*<0.0001) respiratory complications (OR: 3.91, 95% CI: 2.96–5.17, *P*<0.0001), longer hospital length of stay (mean difference: 2.69, 95% CI: 1.68–3.69, *P*<0.0001), and myocardial infarction (OR: 1.85, 95% CI: 1.30–2.65, *P*=0.0007).

**Conclusion:**

Postoperative bleeding requiring re-exploration is associated with lower long-term survival and increased risk of short-term adverse events including operative mortality, stroke, renal and respiratory complications, and longer hospital length of stay. To improve both short-term and long-term outcomes, strategies to prevent the need for re-exploration are necessary.

## Introduction

Highlights

- This is the first meta-analysis that analyzes long-term mortality after re-exploration for bleeding.
- Patients who required re-exploration had significantly higher rates of long-term, all-cause mortality compared to patients who did not.
- Patients requiring re-exploration had significantly higher rates of operative mortality, stroke, renal complications, respiratory complications, longer hospital length of stay, and myocardial infarction when compared with patients who did not require re-exploration for bleeding.

Postoperative bleeding requiring re-exploration is a serious but infrequent complication that occurs in 2.8–4.6% of patients undergoing cardiac surgery^1^. The three most frequently reported sites of bleeding are the body of grafts (20.2%), the sternum (17.0%), and vascular sutures (12.5%)^2^.

A prior meta-analysis including 597 923 patients found that re-exploration for bleeding was strongly and significantly associated with a higher risk of operative mortality [risk ratio (RR) 3.27; 95% CI: 2.44–4.37], stroke (RR 2.18; 95% CI: 1.96–2.43), and sternal wound infection (RR 4.52; 95% CI: 3.95–5.18) at short-term follow-up^3^.

While it is known that re-exploration for bleeding is associated with higher risk of operative mortality after cardiac surgery, its association with long-term outcomes in patients undergoing cardiac surgery remains unclear, and published studies that assessed the topic have had contradictory findings^4–9^.

Herein, we performed a systematic review and meta-analysis with the aim of assessing the long-term impact of postoperative bleeding requiring re-exploration among patients undergoing cardiac surgery.

## Material and methods

Ethical approval was waived as no human subjects or animals were involved. This meta-analysis was registered with the Research Registry UIN and PROSPERO, both databases for systematic review protocols. This work is reported in line with Preferred Reporting Items for Systematic Reviews and Meta-Analyses (PRISMA) (Supplemental Digital Content 1, http://links.lww.com/JS9/C716, Supplemental Digital Content 2, http://links.lww.com/JS9/C717) and Assessing the Methodological Quality of Systematic Reviews (AMSTAR) (Supplemental Digital Content 3, http://links.lww.com/JS9/C718) Guidelines^10,11^.

### Search strategy

A medical librarian performed a comprehensive literature search to identify studies reporting long-term outcomes in patients who required re-exploration for bleeding after cardiac surgery versus patients who did not, with at least 1-year of follow-up. Three databases were queried: Ovid MEDLINE (1946 to present), Ovid EMBASE (1974 to present), and the Cochrane Library. The search strategy for Ovid MEDLINE is available in Supplementary Table 1 (Supplemental Digital Content 4, http://links.lww.com/JS9/C719).

### Study selection and data extraction

Studies were screened by two different reviewers and discrepancies were resolved by the senior author. A first round of screening based on title and abstract content was performed. Studies were considered for inclusion if they were written in English and compared long-term outcomes in patients who required re-exploration for bleeding after cardiac surgery versus patient who did not, with at least 1-year follow-up. Studies not reporting long-term outcomes, abstracts, case reports, commentaries, editorials, expert opinions, conference presentations, and animal studies were excluded. For the second round of screening, the full text of the selected studies was pulled. References lists were also reviewed for relevant studies not initially captured.

Two investigators independently performed data extraction, while its accuracy was verified by the corresponding author. From each study, the following variables were extracted: study characteristics (publication year, country, sample size, study design, mean follow-up, and type of surgery) as well as patient demographics and clinical data [age, sex, BMI, smoking status, hypertension, diabetes, chronic obstructive pulmonary disease (COPD), prior cerebrovascular accident (CVA), prior myocardial infarction (MI), cardiopulmonary bypass, and cross-clamp time]. The quality of the included studies was assessed using the Newcastle–Ottawa Scale for observational studies (Supplementary Table 2, Supplemental Digital Content 4, http://links.lww.com/JS9/C719).

### Outcomes

The primary outcome was long-term all-cause mortality. Secondary outcomes were operative all-cause mortality, stroke, renal, and respiratory complications, MI, and hospital length of stay. The specific definition of the endpoints is provided in Supplementary Table 3 (Supplemental Digital Content 4, http://links.lww.com/JS9/C719).

### Statistical analysis

Categorical variables were analyzed using odds ratio (OR), incidence rate ratio (IRR), and 95% CI. An OR and IRR greater than 1 indicated that an outcome was more frequently present in the re-explored arm. Continuous variables were analyzed using mean difference (MD) and 95% CI. A MD lower than zero corresponded to larger values in the re-explored arm.

Clinical heterogeneity between studies was assessed by random effects models. Results were displayed using forest plots. Between-study statistical heterogeneity was assessed with the Cochran *Q* statistic and by estimating *I* ^2^. High heterogeneity was confirmed with a significance level of *P*<0.10 and *I* ^2^ of at least 50% or more. Subgroup analyses of the primary outcome were performed based on types of surgery, criteria for re-exploration and single versus multicenter studies to investigate heterogeneity.

### Reconstruction of individual patient survival data

The methods described by Wei *et al*. were used to reconstruct individual patient data from the Kaplan–Meier curves of all eligible studies for the long-term outcome^12,13^. Raster and Vector images of the Kaplan–Meier survival curves were preprocessed and digitized, so that the values reflecting specific timepoints and their corresponding survival/mortality information could be extracted. Where additional information (e.g. number-at-risk tables or total number of events) was available, it was used to further calibrate the accuracy of the time-to-events. Departures from monotonicity were detected using isotonic regression and corrected with a pool-adjacent-violators algorithm^12,13^. To confirm the quality of the timing of failure events captured, we thoroughly checked the consistency with the reported survival or mortality data provided in the original publications.

### Meta-analysis of reconstructed data - One-stage survival meta-analysis

The Kaplan–Meier method was used to calculate the overall survival. The Cox proportional hazards regression model was used to assess differences between groups. For these Cox models, the proportional hazards assumption was verified by plotting scaled Schoenfeld residuals, log–log survival plots, and predicted versus observed survival functions. The survival curves were plotted using the Kaplan–Meier product limit method and hazard ratios (HRs) as well as 95% CIs for each group were calculated.

### Sensitivity analyses

Leave-one-out analysis for the primary outcome was performed to assess the robustness of the obtained estimate. A funnel plot was performed to assess publication bias. Meta-regression was used to explore the effects of sex, BMI, smoking status, hypertension, diabetes, COPD, prior CVA, prior MI and chronic kidney disease. All statistical analyses were performed using R (version 4.3.1, R Project for Statistical Computing) within RStudio and STATA IC17.0 (StataCorp LLC, College Station, Texas).

## Results

A total of 2583 studies were retrieved from the systematic search, six of which met the inclusion criteria. The PRISMA flow diagram outlining the study selection process is provided in Supplementary Figure 1 (Supplemental Digital Content 4, http://links.lww.com/JS9/C719).

### Study characteristics

The included studies were published between 2021 and 2023 and were all observational. The included studies originated from United States, Sweden, Poland, Iceland, Denmark, or the Netherlands. A total of 135 456 patients were included in the final analysis. The number of patients in each study ranged from 2060 to 48 060 with a median sample size of 16 579.5 [interquartile range (IQR): 30 529 [10 824–41 353]) (Table 1).

**Table 1:** Summary of included studies.

References	Year of publication	Country	No. of patients	Study design	Mean follow-up (years)	Type of surgery
Brown^7^	2020	United States	10 824	Single-center, retrospective	4.0	CABG, valve replacement/repair or combined procedure
Heimisdottir^8^	2022	Sweden	48 060	Multicenter, retrospective	4.6	CABG, valve replacement/repair or combined procedure
Knapik^9^	2019	Poland	41 353	Multicenter, retrospective	2.6	Isolated CABG
Marteisson^10^	2020	Iceland	2060	Multicenter, retrospective	7.6	Isolated CABG
Qazi^11^	2021	Denmark	11 813	Single-center, retrospective	12.5	CABG, valve replacement/repair or combined procedure
Stroo^12^	2023	Netherlands	21 346	Single-center, retrospective	9.7	Isolated CABG

CABG, coronary artery bypass grafting.

### Patient characteristics

Demographic data of each study’s patient population is summarized in Supplementary Table 4 (Supplemental Digital Content 4, http://links.lww.com/JS9/C719). Age ranged from 64.9 to 70.3 years; the percentage of female patients ranged from 13.9 to 31.3%; the percentage of actively smoking patients ranged from 13.4 to 73.1%; the percentage of patients with hypertension ranged from 36.9 to 88.5%; the percentage of patients with diabetes ranged from 11.2 to 85.3%; the percentage of patients with COPD ranged from 3.5 to 12.2%; the percentage of patients with prior CVA ranged from 3.6 to 23.6%; the percentage of patients with prior MI ranged from 0.9 to 34.6%; the cardiopulmonary bypass time ranged from 87 to 132 min; the cross-clamp time ranged from 46 to 67 min. Criteria used for re-exploration are summarized in Supplementary Table 5 (Supplemental Digital Content 4, http://links.lww.com/JS9/C719).

### Primary outcome

#### Meta-analysis of reconstructed data - One-stage survival meta-analysis

**Figure 1:**
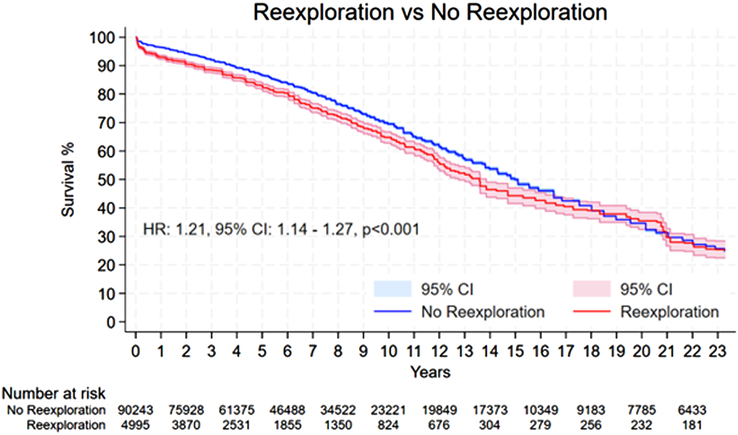
Pooled Kaplan–Meier curve showing the cumulative risk of all-cause mortality following re-exploration for bleeding versus no re-exploration. HR, hazard ratio.

Patients who underwent re-exploration had significantly higher risk of death during follow-up compared to the patients who did not (HR: 1.21, 95% CI: 1.14–1.27, *P*<0.001) (Fig. 1).

**Figure 2:**
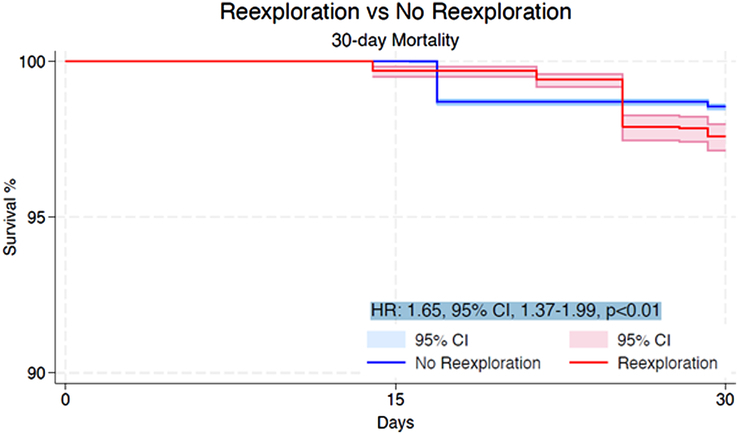
Pooled Kaplan–Meier curve showing the cumulative risk of all-cause mortality following re-exploration for bleeding versus no re-exploration at 30 day follow-up. HR, hazard ratio.

**Figure 3:**
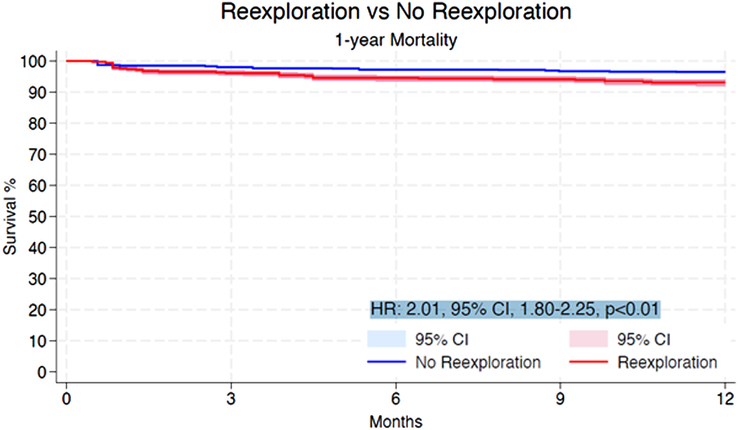
Pooled Kaplan–Meier curve showing the cumulative risk of all-cause mortality following re-exploration for bleeding versus no re-exploration at 1-year follow-up. HR, hazard ratio.

**Figure 4:**
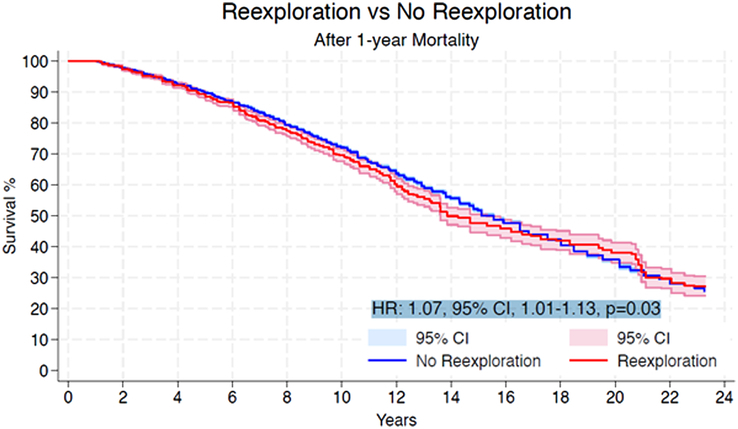
Pooled Kaplan–Meier curve showing the cumulative risk of all-cause mortality following re-exploration for bleeding versus no re-exploration beyond 1-year follow-up. HR, hazard ratio.

Within the initial 30 days, the re-exploration cohort experienced a higher incidence of mortality in comparison to the non re-explored group (HR: 1.65, 95% CI: 1.37–1.99, *P*<0.01) (Fig. 2). At the 1-year mark, the mortality rate was also significantly higher for patients who underwent re-exploration than for those who did not (HR: 2.01, 95% CI: 1.80–2.25, *P*<0.01) (Fig. 3). Beyond 1-year, patients subjected to re-exploration showed a statistically significant, albeit modest, increase in mortality compared to those who did not undergo re-exploration for bleeding (HR: 1.07, 95% CI: 1.01–1.13, *P*=0.03) (Fig. 4).

#### Two-stage survival meta-analysis

**Figure 5:**
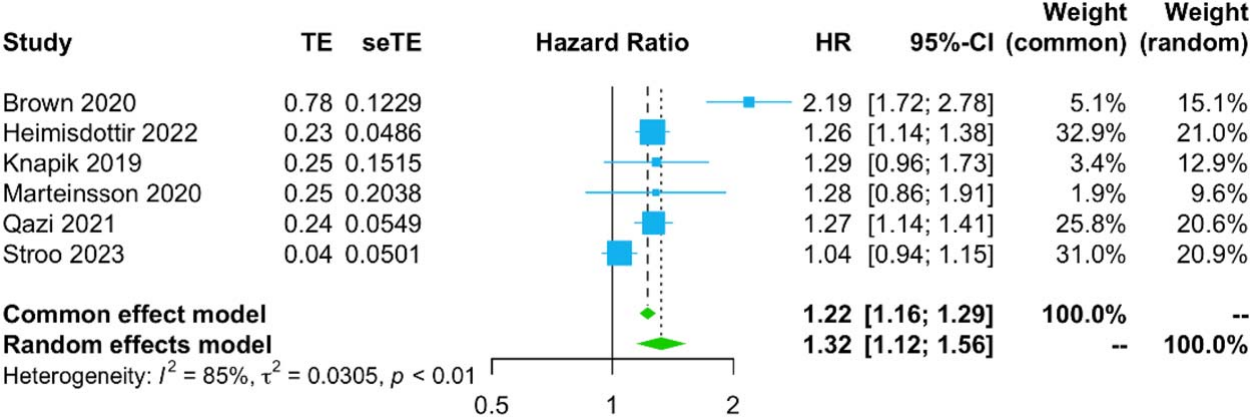
Forest plot for the primary endpoint (long-term all-cause mortality). HR, hazard ratio.

Figure 5 shows the forest plot for long-term all-cause mortality. Patients who required re-exploration had significantly higher rates of long-term all-cause mortality compared to patients who did not (HR: 1.32, 95% CI: 1.12–1.56, *P*<0.01). The subgroup analyses was qualitatively consistent with the primary analysis (Supplementary Figures 9-11, Supplemental Digital Content 4, http://links.lww.com/JS9/C719).

#### Sensitivity analyses

The leave-one-out analysis for the primary outcome showed slight variations in the pooled IRR (Supplementary Figure 2A, Supplemental Digital Content 4, http://links.lww.com/JS9/C719). The funnel plot showed no evidence of publication bias (Egger’s test: 0.167±2.268, *P*=0.955 – Supplementary Figure 2B, Supplemental Digital Content 4, http://links.lww.com/JS9/C719).

In the meta-regression analysis, hypertension was associated with higher IRRs for the primary outcome (Beta= 0.0137±0.0063, *P*=0.03 – Supplementary Table 6, Supplemental Digital Content 4, http://links.lww.com/JS9/C719).

### Secondary outcomes

Table 2 summarizes other key results of the meta-analysis including the investigated secondary outcomes and their respective effect estimates.

**Table 2:** Outcomes summary.

Outcome	Number of studies	Number of patients	Effect estimate (95% CI, *P*)
Long-term all-cause mortality	6	95 238 135 057	IPD Analysis: HR: 1.21, 95% CI: 1.14–1.27, *P*<0.001 Two-stage Analysis: HR: 1.32, 95% CI: 1.12–1.56, *P*<0.01
Operative all-cause mortality	6	135 057	OR: 5.25, 95% CI: 4.74–5.82, *P*<0.0001
Stroke	6	135 057	OR: 2.05, 95% CI: 1.72–2.43, *P*<0.0001
Renal complications	5	114 110	OR: 4.13, 95% CI: 3.43–4.39 *P*<0.0001
Respiratory complications	5	114 110	OR: 3.91, 95% CI: 2.96–5.17, *P*<0.0001
Hospital length of stay	5	93 704	SMD: 0.64, 95% CI: 0.50–0.78, *P*<0.0001
Myocardial infarction	3	34 820	OR: 1.85, 95% CI: 1.30–2.65, *P*=0.0007

HR, hazard ratio; IPD, individual patient data; OR, odds ratio; SMD, standard mean difference.

Patients requiring re-exploration had significantly higher rates of operative, all-cause mortality compared to patients who did not (OR: 5.25, 95% CI: 4.74–5.82, *P*<0.0001) (Supplementary Figure 3, Supplemental Digital Content 4, http://links.lww.com/JS9/C719). Patients requiring re-exploration had significantly higher rates of stroke (OR: 2.05, 95% CI: 1.72–2.43, *P*<0.0001), renal complications (OR: 4.13, 95% CI: 3.43–4.39 *P*<0.0001), respiratory complications (OR: 3.91, 95% CI: 2.96–5.17, *P*<0.0001), longer hospital length of stay (MD: 2.69, 95% CI: 1.68–3.69, *P*<0.0001), and MI (OR: 1.85, 95% CI: 1.30–2.65, *P*=0.0007) when compared with patients who did not require re-exploration for bleeding (Supplementary Figures 4-8, Supplemental Digital Content 4, http://links.lww.com/JS9/C719).

## Discussion

This meta-analysis of six studies and 135 456 cardiac surgery patients found that patients undergoing re-exploration for bleeding have a higher risk of long-term mortality compared with those who did not undergo re-exploration, a finding which was consistent in all the sensitivity analyses. Re-exploration for bleeding was also associated with increased risk of operative mortality, stroke, myocardial infraction, renal complications, respiratory complications, and prolonged hospital length of stay.

This is the first meta-analysis that analyzes long-term mortality after re-exploration for bleeding. A prior meta-analysis by Biancari *et al*. showed that re-exploration performed more than 12 h after cardiac surgery was associated with a significantly higher risk of postoperative mortality [risk ratio (RR): 3.27, 95% CI: 2.44–4.37, *P*<0.00001], stroke (RR: 2.18, 95% CI: 1.96–2.43, *P*<0.00001), intra-aortic balloon pump requirement (RR: 3.34, 95% CI: 1.95–5.72, *P*<0.00001), acute renal failure (RR: 3.70, 95% CI: 2.91–4.69, *P*<0.00001), sternal wound infection (RR: 4.52, 95% CI: 3.95–5.18, *P*<0.00001), and prolonged mechanical ventilation (RR: 3.39, 95% CI: 2.28–5.05, *P*<0.00001)^3^.

While risk factors and early outcomes of patients undergoing re-exploration have been studied, research on the mid-term and long-term outcomes is limited and mixed^7,9^. Three of the six published studies found no significant difference in long-term mortality between patients who did and did not undergo re-exploration, while the remaining studies reported higher short-term mortality among patients who underwent re-exploration^4,6,9^. The studies that reported no significant difference between re-exploration and no-re-exploration had longer follow-up (Heimisdottir: mean 4.6 years, Marteisson: median 7.6 years, Stroo: median 9.7 years)^4,6,9^.

Our study has corroborated previous short-term results, bringing new evidence concerning long-term outcomes of re-exploration for bleeding after cardiac surgery.

Our data suggests that to reduce morbidity and mortality after cardiac surgery, critical preventive and procedural measures need to be established in order to avoid re-exploration. A comprehensive approach should be considered addressing preoperative, intraoperative, and postoperative practices^14^. Preoperatively, it is important to identify patients with high risk of bleeding by conducting a thorough preoperative history^15^. Risk assessment tools like FMT and other preoperative risk assessment tools could be used to aid assessment of bleeding risk^12^. Intraoperatively, preventive measures such as acute normovolemic hemodilution, intraoperative cell salvage, ultrafiltration, pharmacological therapies targeting hemostasis, coagulation, and fibrinolysis could help reduce bleeding^15^. Intraoperative checklists may also be useful to predict postoperative bleeding^15^. Postoperatively, avoiding iatrogenic blood loss and utilizing re-exploration only when necessary are essential^15^. In fact, conservative management for stable patients with significant but noncritical bleeding might be a safe option, thereby reducing postoperative morbidity and hospital stay^16^.

The unfavorable outcomes after re-exploration for bleeding are likely multifactorial. Identifying a single underlying cause for these poor outcomes is challenging. Further research is needed to better understand the mechanisms involved while continuing to explore strategies for improving patient outcomes.

### Study strengths and limitations

This is the first meta-analysis to examine the relationship of re-exploration for bleeding after cardiac surgery and long-term outcomes. Moreover, we assessed seven different outcomes and performed different sensitivity analyses including a meta-regression of nine different preoperative factors.

However, this work has the intrinsic limitations typical of observational studies, including the risk of methodological heterogeneity, residual confounders, and ecologic fallacy of meta-regression. Most importantly, criteria for re-exploration and the definition of secondary outcomes were heterogenous and/or poorly defined across studies Table 3.

**Table 3:** Criteria and timing of re-exploration.

References	Criteria for re-exploration	Timing of re-exploration
Brown^7^	• Hemodynamic instability that accompanied excessive bleeding, declining hemoglobin, or radiographic evidence of mediastinal hematoma. • Excessive bleeding greater than 800 ml in the first hour after arrival to the ICU or greater than 300 ml in each of the first 4 h after arrival to the ICU. • Moderate, but continuous, bleeding that was persistently greater than 150 ml per hour, usually for more than 12 h. • Signs of cardiac tamponade. • Radiographic findings of mediastinal hematoma on routine postoperative imaging, along with declining hemoglobin, but without signs of hemodynamic instability or excessive bleeding	• ≤24 h after arriving to the ICU: 238 (81.5%) • >24 h after arriving to the ICU54 (18.5)
Heimisdottir^4^	The decision to re-explore the patient was under the discretion of the attending surgeon	Only re-exploration for excessive bleeding or tamponade in the first 24 h from the primary operation was included
Knapik^5^	NR	NR
Marteisson^10^	The decision to reoperate was at the discretion of the attending surgeon	NR
Qazi^8^	This center adheres to the Kirklin/Barrat-Boyes criteria for reoperation or on the type of bleeding such as sudden massive bleeding, or hemodynamic instability. Reoperation is initiated if drainage output is: • >500 ml during the first hour • >400 ml during each of the first 2 h • >300 ml during each of the first 3 h • >1000 ml in total in the first 4 h	NR
Stroo^9^	The decision to perform re-exploration was made by the cardiac surgeon and was based on these criteria: Drainage of: •>500 ml during the first hour • >400 ml during each of the first 2 h •>300 ml during each of the first 3 h • >1000 ml in total in the first 4 h. • Continued bleeding despite correction of coagulopathies • Instant massive bleeding	NR

## Conclusion

Re-exploration for bleeding after cardiac surgery is associated with a higher risk of long-term mortality, operative mortality, and morbidity after cardiac surgery. These findings highlight the substantial impact of re-exploration for bleeding on both short-term and long-term outcomes following adult cardiac surgery.

## Ethical approval

Ethical approval waived as no human subjects or animals were involved.

## Consent

None.

## Source of funding

None.

## Author contribution

G.J.S.: conceptualization, methodology, supervision, writing – original draft, and writing – review and editing; G.C.: conceptualization, data curation, writing – original draft, and writing – review and editing; M.D.A.: conceptualization, data curation, and writing – original draft; T.C.: formal analysis; L.H.: supervision, writing – original draft, and writing – review and editing; C.R.: data curation and writing – review and editing; P.T.: formal analysis, investigation, methodology, and supervision; J.L.: writing – original draft and writing – review and editing; K.R.A.: formal analysis and writing – review and editing; A.D.: conceptualization, formal analysis, and writing – review and editing; M.D.: data curation; M.G.: conceptualization, methodology, supervision, validation, writing – original draft, and writing – review and editing.

## Conflicts of interest disclosure

The authors declare that they have no financial conflict of interest with regard to the content of this report.

## Research registration unique identifying number (UIN)

Research Registry UIN ID # reviewregistry1827 PROSPERO ID # CRD42023446906.

## Guarantor

Mario Gaudino MD PhD.

## Data availability statement

The data underlying this article are available in the article and in its online supplementary material. The current study data are available upon reasonable request to the corresponding author.

## Provenance and peer review

Not commissioned, externally peer-reviewed.

## Disclosure

None.

## Supplementary Material

**Figure:**