# Supplementary material for: Re-exploration for bleeding and long-term survival after adult cardiac surgery: a meta-analysis of reconstructed time-to-event data
Source: Int J Surg. 2024 Jun 7;110(9):5795–801. doi: 10.1097/JS9.0000000000001765 (PMC11392166; doi:10.1097/JS9.0000000000001765)
Supplement: Supplementary file 3 [file js9-110-5795-s004.docx]

**Supplementary Material**

**Supplementary Table 1.** Search strategy for Ovid MEDLINE.

**Supplementary Table 2.** Newcastle-Ottawa Scale.

**Supplementary Table 3.** Primary and secondary outcome definitions.

**Supplementary Table 4.** Demographics of included patients from included studies.

**Supplementary Table 5.** Meta-regression of clinical variables regarding the primary endpoint (long-term all-cause mortality).

**Supplementary Figure 1.** Preferred Reporting Items for Systematic Reviews and Meta-Analyses (PRISMA) flow diagram.

**Supplementary Figure 2:** Sensitive analyses for the primary endpoint (long-term all-cause mortality): leave-one-out analysis (A) and funnel plot (B).

**Supplementary Figure 3.** Forest plot for operative mortality.

**Supplementary Figure 4.** Forest plot for stroke.

**Supplementary Figure 5**. Forest plot for renal complications.

**Supplementary Figure 6**. Forest plot for respiratory complications.

**Supplementary Figure 7**. Forest plot for hospital length of stay.

**Supplementary Figure 8**. Forest plot for myocardial infarction.

**Supplementary Figure 9.** Subgroup analysis of primary outcome based on types of surgery.

**Supplementary Figure 10.** Subgroup analysis of primary outcome based on criteria for re-exploration.

**Supplementary Figure 11.** Subgroup analysis of primary outcome based on single versus multi-centers studies.

**Supplementary Table 1.** Search strategy for Ovid MEDLINE (part 1).

| **Line #** | **Search** |
| --- | --- |
| 1 | Reoperation/ |
| 2 | (reoperat* or re-operat* or re-explor*).tw. |
| 3 | ((repeat* or revision or second) adj3 (surg* or operat* or procedure*)).tw. |
| 4 | or/1-3 |
| 5 | Postoperative Hemorrhage/ |
| 6 | ((bleed* or hemorrhag* or blood loss) adj3 (following or after or post*)).tw. |
| 7 | 5 or 6 |
| 8 | Cardiac Surgical Procedures/ |
| 9 | (cardiac surgery or cardiac surgical procedure* or heart surgery or heart valve surgery or heart surgical procedures* or cardiac operation* or heart operation* or cardiosurgery or myocardial resection).tw. |
| 10 | Coronary Artery Bypass/ or Coronary Artery Bypass, Off-Pump/ |
| 11 | (coronary adj2 (bypass* or graft* or surger*)).tw. |
| 12 | (CABG or aorticocoronary anastomosis or total arterial revasculari*ation* or multiple arterial revasculari*ation*).tw. |
| 13 | Heart Transplantation/ |
| 14 | (heart transplant* or heart graft* or cardiac transplant*).tw. |
| 15 | Cardiomyoplasty/ or (cardiomyoplasty or cardiomyoplasties).tw. |
| 16 | Fontan Procedure/ or (Fontan procedure or "Stage 3 Norwood procedure" or "Stage III Norwood procedure" or "Stage 2 Norwood Procedure" or "Stage II Norwood Procedure" or bidirectional Glenn or bidirectional cavopulmonary shunt).tw. |
| 17 | Heart Valve Prosthesis Implantation/ or (heart valve prosthesis implantation or heart valve prosthesis implant).tw. |
| 18 | Myocardial Revascularization/ or (cardiac muscle revascularisation or cardiac muscle revascularization or coronary revascularisation or coronary revascularization or heart muscle revascularisation or heart myocardium revascularisation or heart revascularisation or heart revascularization or internal mammary arterial anastomosis or internal mammary arterial implantation or internal mammary artery anastomosis or internal mammary artery graft or internal mammary artery implant or internal mammary artery implantation or internal mammary-coronary artery anastomosis or Coronary Internal Mammary Artery Anastomosis or myocardial revascularisation or myocardial revascularization or myocardium revascularisation or myocardium revascularization or transmyocardial laser revascularisation or transmyocardial laser revascularization or vineberg operation).tw. |
| 19 | Cardiac Valve Annuloplasty/ or (Cardiac Valve Annuloplasty or Cardiac Valve Annuloplasties or Valvular Annuloplasties or Valvular Annuloplasty or Heart Valve Annuloplasty or Heart Valve Annuloplasties or Cardiac Valve Annulus Repair or Heart Valve Annulus Repair or Cardiac Valve Annular Repair or Heart Valve Annular Repair or Cardiac Valve Annular Reduction or Cardiac Valve Annulus Shortening or Cardiac Valve Annulus Reduction).tw. |
| 20 | (Aortic Valve Repair or Aortic Valve Replacement or aorta valve replacement or aorta valve transplantation or aortic valve transplantation or aortic valve xenotransplantation).tw. |
| 21 | (tricuspid valve repair or tricuspid valve replacement or tricuspid valve transplantation).tw. |

**Supplementary Table 1.** Search strategy for Ovid MEDLINE (part 2).

| 22 | Heart-Assist Devices/ or (heart assist device* or heart assist pump* or vascular assist device* or artificial ventricle* or ventricle assist device* or artificial heart ventricle*).tw. |
| --- | --- |
| 23 | Transmyocardial Laser Revascularization/ |
| 24 | (transmyocardial laser revascularization or trans-myocardial laser revascularization or transmyocardial laser revascularisation or trans-myocardial laser revascularisation).tw. |
| 25 | Norwood Procedures/ or (Norwood procedure* or Norwood operation*).tw. |
| 26 | Arterial Switch Operation/ |
| 27 | (arterial switch or atrial switch or double switch technique* or Rastelli operation or Rastelli procedure or Rastelli technique or Senning operation or Senning procedure or Jatene procedure or Jatene technique or Jatene operation or Mustard operation or Mustard repair or Mustard procedure).tw. |
| 28 | Mitral Valve Annuloplasty/ |
| 29 | ((bicuspid cardiac valve or bicuspid cardiac valvular or bicuspid heart valve or bicuspid heart valvular or bicuspid or bicuspid valve or bicuspid valvular or left atrioventricular cardiac valve or left atrioventricular heart valve or left atrioventricular valvular or mitral cardiac valve or mitral cardiac valvular or mitral heart valve or mitral heart valvular or mitral or mitral valvular) adj2 (annuloplast* or repair or replacement)).tw. |
| 30 | Pericardial Window Techniques/ or (pericardial window or pericardiostomy or pericardiostomies).tw. |
| 31 | Pericardiectomy/ or (pericardiectomy or pericardiectomies or pericardectomy or pericardectomies or pericardiotomy or pericardiotomies or pericardotomy or pericardotomies).tw. |
| 32 | or/8-31 |
| 33 | 4 and 7 and 32 |

**Supplementary Table 2.** Newcastle-Ottawa Scale.

| Study, year | Selection | Comparability | Outcome |
| --- | --- | --- | --- |
| Brown, 2020 | **** | ** | *** |
| Heimsdottir, 2022 | **** | ** | *** |
| Kanpik, 2019 | **** | ** | *** |
| Marteinsson. 2020 | **** | ** | *** |
| Qazi, 2021 | **** | ** | *** |
| Stroo, 2023 | **** | ** | *** |

**Supplementary Table 3.** Primary and secondary outcomes definitions.

| Study, year | Long term mortality | Operative mortality | Stroke | Renal complications | Respiratory complications | Myocardial Infarction |
| --- | --- | --- | --- | --- | --- | --- |
| Brown, 2020 | Death occurred after hospital discharge / 30 days after the operation | Death occurred before hospital discharge or within 30 days of the operation | Not defined | New dialysis requirement | Mechanical ventilation >24h | Not reported |
| Heimsdottir, 2022 | Death occurring >90 days postoperatively  (Until the end of follow-up) with survival time being  counted from the date of the operation | Not defined | Not defined | Postoperative need for dialysis | Postoperative need for mechanical ventilation | Not reported |
| Kanpik, 2019 | Not defined | Not defined | Not defined | Not defined | Not defined | Not reported |
| Marteinsson. 2020 | Death after 30 days following the primary operation | Death within 30 days following the primary operation | Neurological signs that persisted for more than 24 h. | Postoperative renal failure | Pneumonia and pleural effusion | Isolated ST-segment changes or a new left bundle branch block on electrocardiogram, along with elevation of creatine kinase MB of >70 lg/l |
| Qazi, 2021 | Not defined | Not defined | Not defined | Postoperative need for dialysis | Mechanical ventilation >48h | Not defined |
| Stroo, 2023 | Death after 30 days following the primary operation to the end of the follow-up | Death occurred before hospital discharge or within 30 days of the operation | Acute onset of clinical symptoms due to cerebral infarction or hemorrhage on neurologic imaging (either CT or MRI), with symptoms persistent for >24 hours with or without residual deficit or until death. | Not reported | Not reported | Not defined |

**Supplementary Table 4.** Patient demographics from the selected studies.

| Study | Age  (mean±SD) | | Female (%) | | Smoking  (%) | | | HTN (%) | | DM (%) | | | COPD  (%) | | Prior CVA (%) | | | Prior MI (%) | | | CPB Time  (min) | | | XC time  (min) | |
| --- | --- | --- | --- | --- | --- | --- | --- | --- | --- | --- | --- | --- | --- | --- | --- | --- | --- | --- | --- | --- | --- | --- | --- | --- | --- |
|  | **RE** | **NRE** | **RE** | **NRE** | **RE** | **NRE** | **RE** | | **NRE** | **RE** | **NRE** | **RE** | | **NRE** | **RE** | **NRE** | **RE** | | **NRE** | **RE** | | **NRE** | **RE** | | **NRE** |
| Brown | 67.4±12.1 | 66.7±11.2 | 28.8 | 31.3 | NR | NR | 82.2 | | 85.3 | 82.2 | 85.3 | 4.8 | | 3.5 | 23.6 | 21.4 | NR | | NR | 132.7±64.9 | | 114.8±47.6 | NR | | NR |
| Heimsdottir | 70.3±10.4 | 69±10.4 | 22.9 | 26.0 | NR | NR | 55.6 | | 55.4 | 21.9 | 23.7 | NR | | NR | 11.1 | 9.9 | 32.9 | | 30.3 | NR | | NR | NR | | NR |
| Kanpik | NR | NR | 21.1 | 25.7 | 13.4 | 16.3 | 88.5 | | 84.6 | 32.3 | 36.2 | 8.8 | | 6.1 | 4.7 | 3.6 | 34.6 | | 27.3 | NR | | NR | NR | | NR |
| Marteinsson | 67.6±9.6 | 66.3±9.3 | 14.6 | 17.6 | 73.1 | 70.5 | 61.5 | | 65.8 | 13.1 | 17.7 | NR | | NR | NR | NR | NR | | NR | 88.6±33.0 | | 87.0±25.2 | 47±15.7 | | 46±15.6 |
| Qazi | 66.9±10.9 | 65.8±10.5 | 25.0 | 25.0 | 17.9 | 19.9 | 40.7 | | 36.9 | 11.2 | 16.8 | NR | | NR | 7.5 | 7.2 | 1.0 | | 0.9 | 105±39 | | 97±36 | NR | | NR |
| Stroo | 64.9±9.9 | 65.0±9.6 | 13.9 | 21.9 | NR | NR | NR | | NR | 18.1 | 22.2 | 12.2 | | 10.3 | 4.5 | 4.5 | 4.9 | | 4.5 | NR | | NR | 67±23 | | 65.8±26 |

CVA=cerebrovascular accident; CPB=cardiopulmonary bypass, DM=diabetes; HTN=hypertension; MI=myocardial infarction;
NR=not reported; NRE=no re-exploration; SD=standard deviation, RE=re-exploration; XC=cross-clamp.

**Supplementary Table 5.** Meta-regression of clinical variables regarding the primary endpoint (long-term all-cause mortality).

| Variables | Beta ± SE, P-value |
| --- | --- |
| Female | -0.0053 ± 0.0054, P=0.3249 |
| BMI | -0.0229 ± 0.1716, P=0.8937 |
| Smoking | -0.0054 ± 0.0114, P=0.6350 |
| Hypertension | 0.0137 ± 0.0063, P=0.0303 |
| Diabetes | 0.0228 ± 0.0124, P=0.0657 |
| COPD | -0.0777 ± 0.1032, P=0.4514 |
| Prior CVA | -0.0011 ± 0.0198, P=0.9560 |
| Prior MI | 0.0072 ± 0.0280, P=0.7975 |
| CKD | 0.0075 ± 0.0327, P=0.8178 |

BMI=body mass index, CKD=chronic kidney diseases, COPD=chronic obstructive pulmonary disease, CVA=cerebrovascular accidents, MI=myocardial infarction.

**Supplementary Figure 1.**  Preferred Reporting Items for Systematic Reviews and Meta-Analyses (PRISMA) flow diagram.


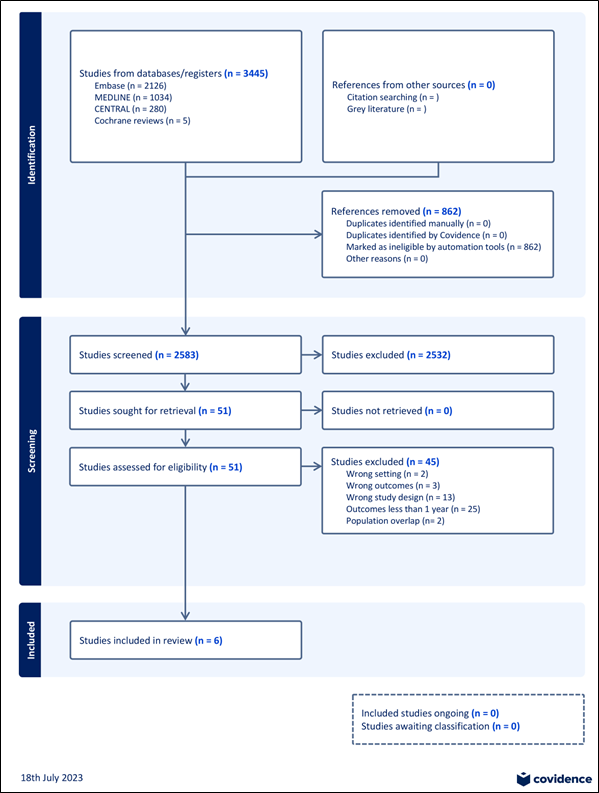


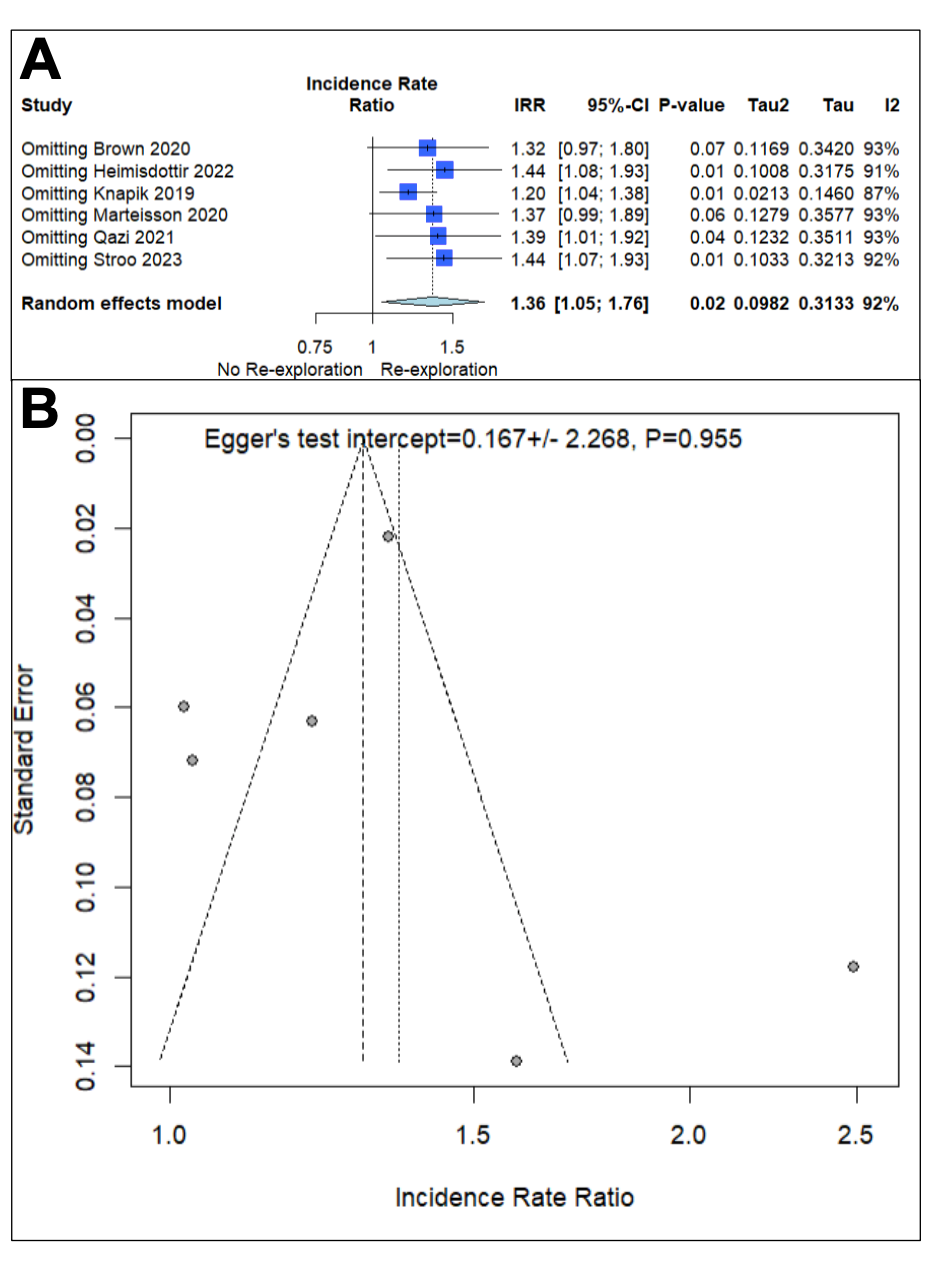
**Supplementary Figure 2:** Sensitivity analyses for the primary outcome (long-term all-cause mortality): leave-one-out analysis (A) and funnel plot (B).

IRR=incidence rate ratio, CI=confidence interval, Tau estimate of the standard deviation of the distribution of true effect sizes, I^2^=heterogeneity level.

**Supplementary Figure 3.** Forest plot for operative mortality.


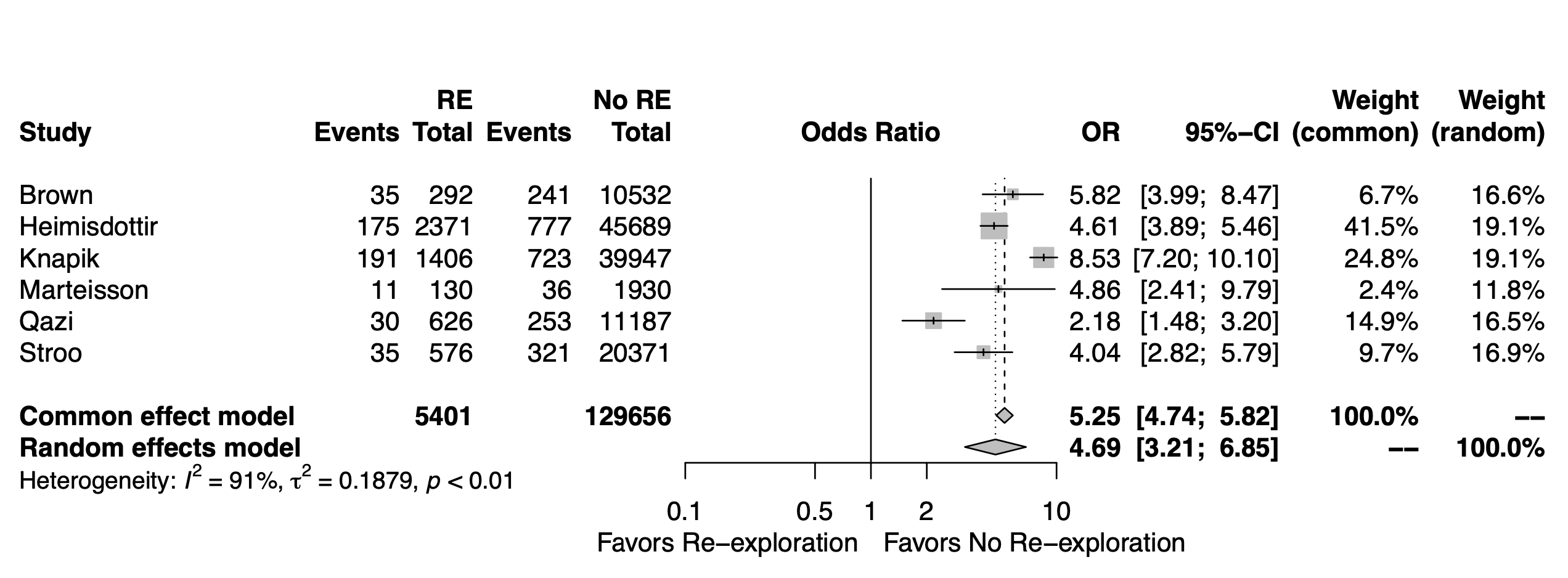


CI=confidence interval, OR=odds ratio, RE=re-exploration.

**Supplementary Figure 4**. Forest plot for stroke.


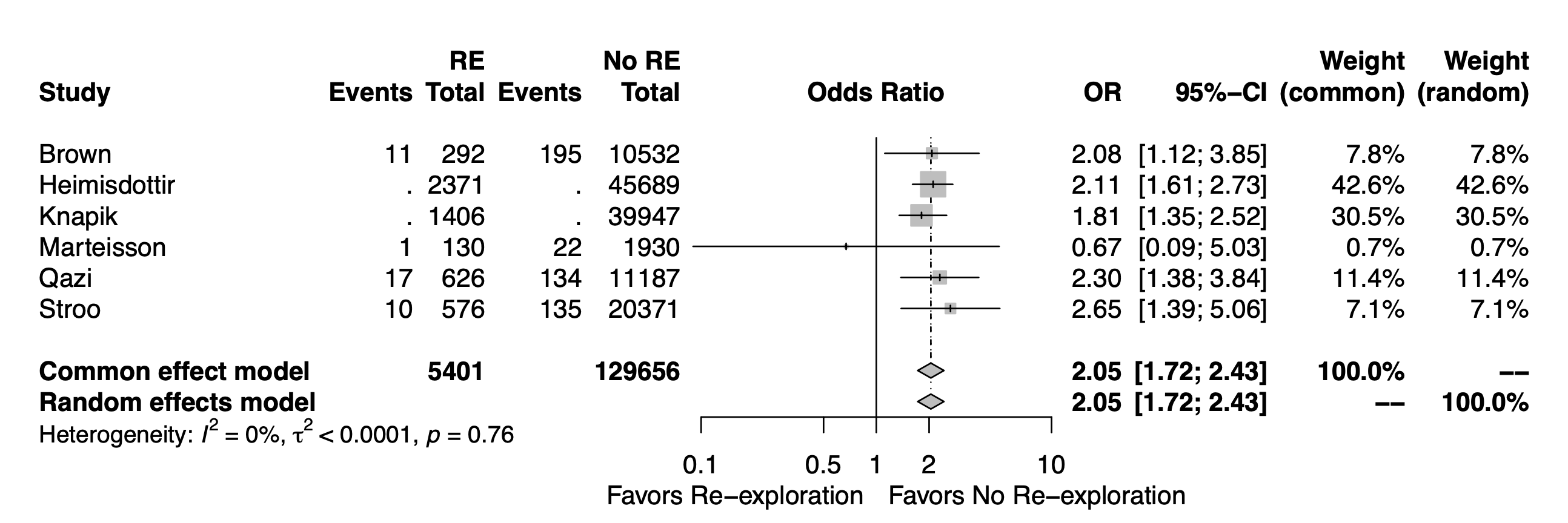


CI=confidence interval, OR=odds ratio, RE=re-exploration.

**Supplementary Figure 5**. Forest plot for renal complications.


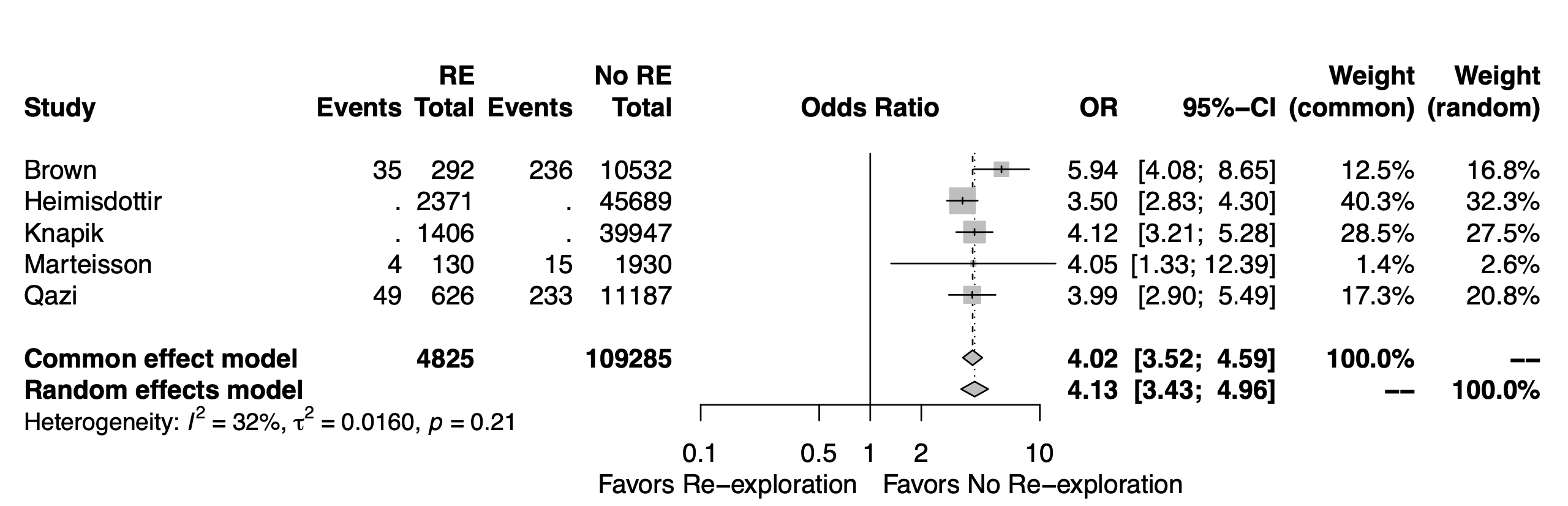


CI=confidence interval, OR=odds ratio, RE=re-exploration.

**Supplementary Figure 6**. Forest plot for respiratory complications.


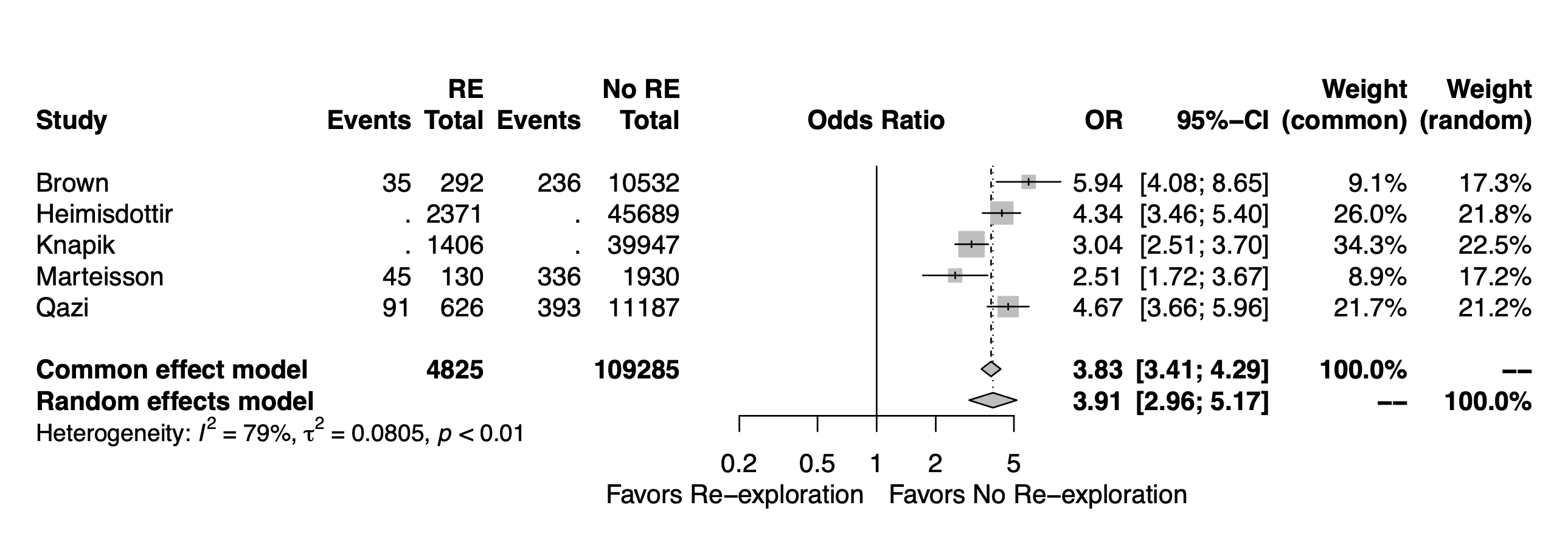


CI=confidence interval, OR=odds ratio, RE=re-exploration.

**Supplementary Figure 7**. Forest plot for hospital length of stay.


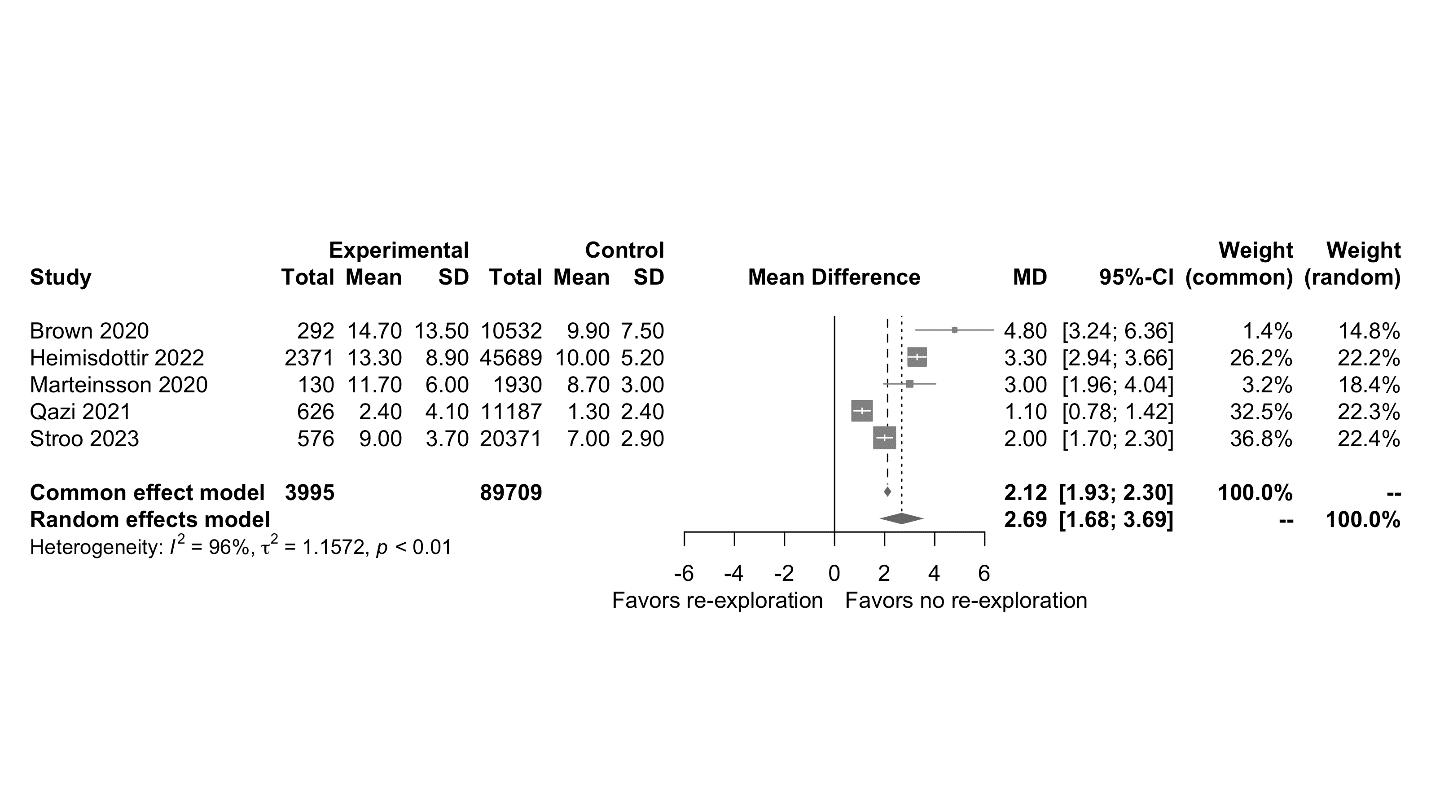


CI=confidence interval, SD=standard deviation, MD= mean difference, RE=re-exploration.

**Supplementary Figure 8**. Forest plot for myocardial infarction.


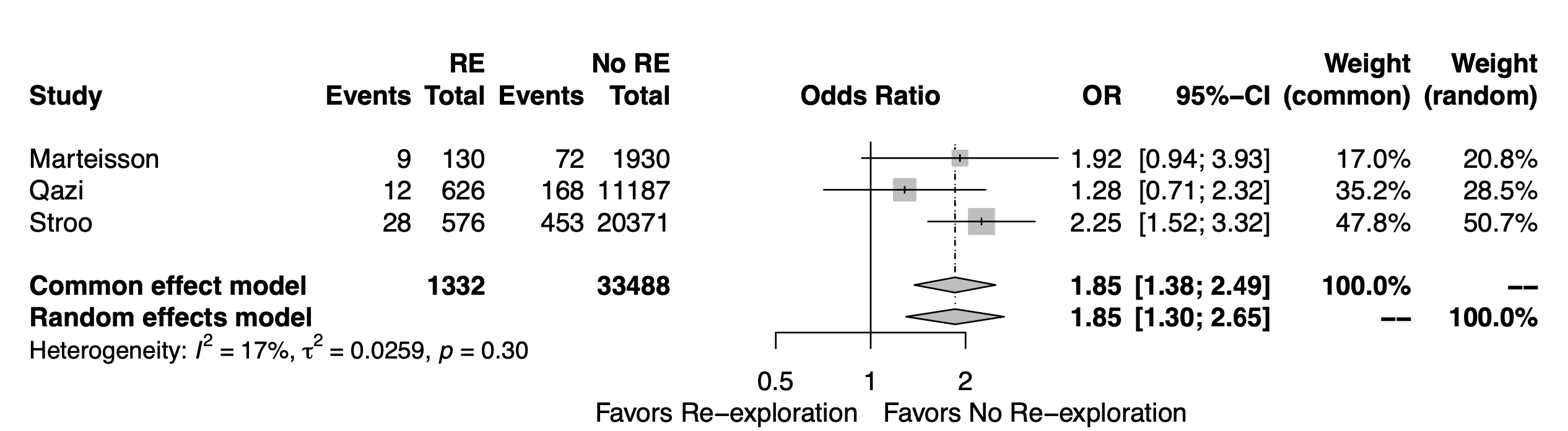


CI=confidence interval, OR=odds ratio, RE=re-exploration.

**Supplementary Figure 9.** Subgroup analysis of primary outcome based on types of surgery.


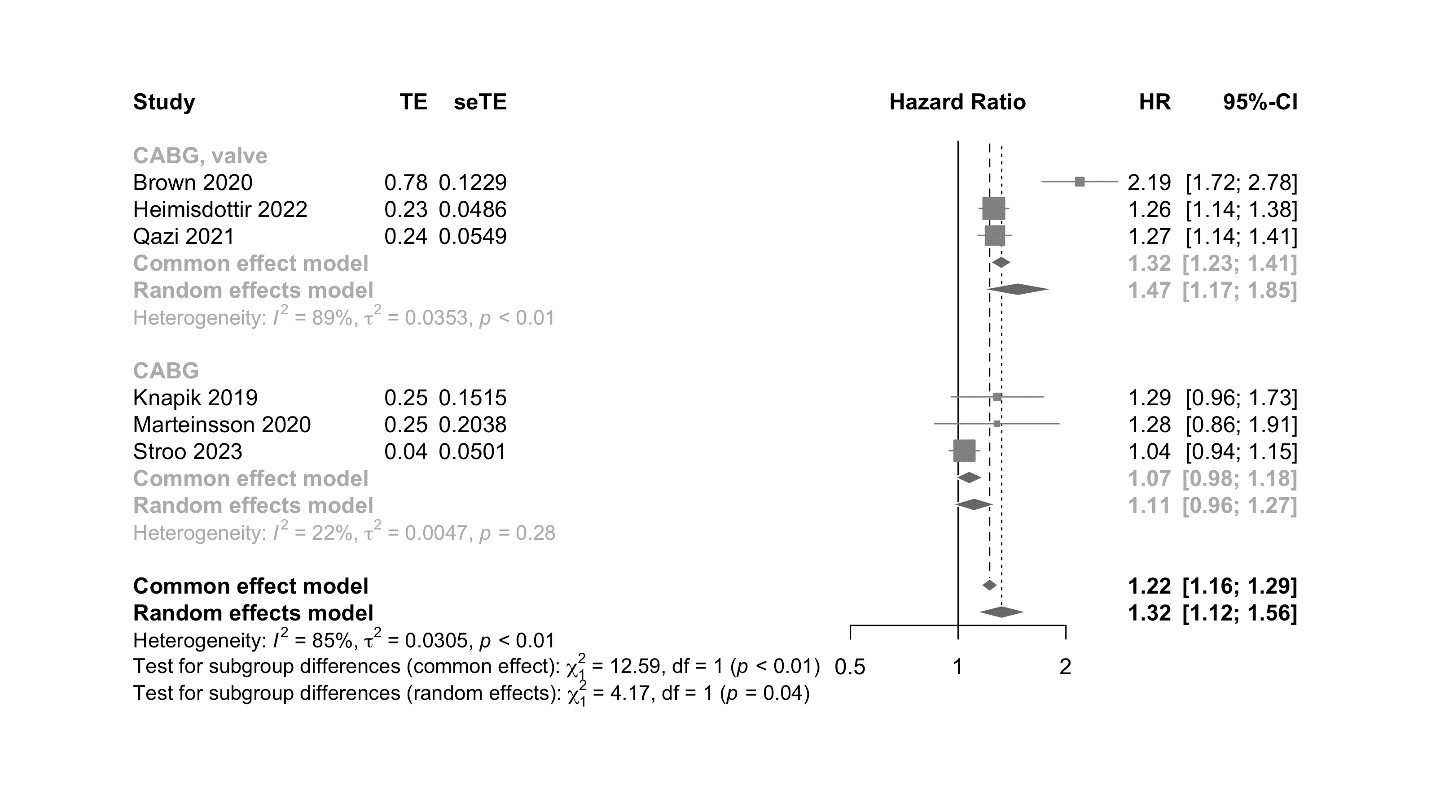


CABG=coronary artery bypass graft, CI=confidence interval, HR=hazard ratio, TE= estimate of treatment effect, seTE=standard error of treatment estimate.

**Supplementary Figure 10.** Subgroup analysis of primary outcome based on criteria for re-exploration.


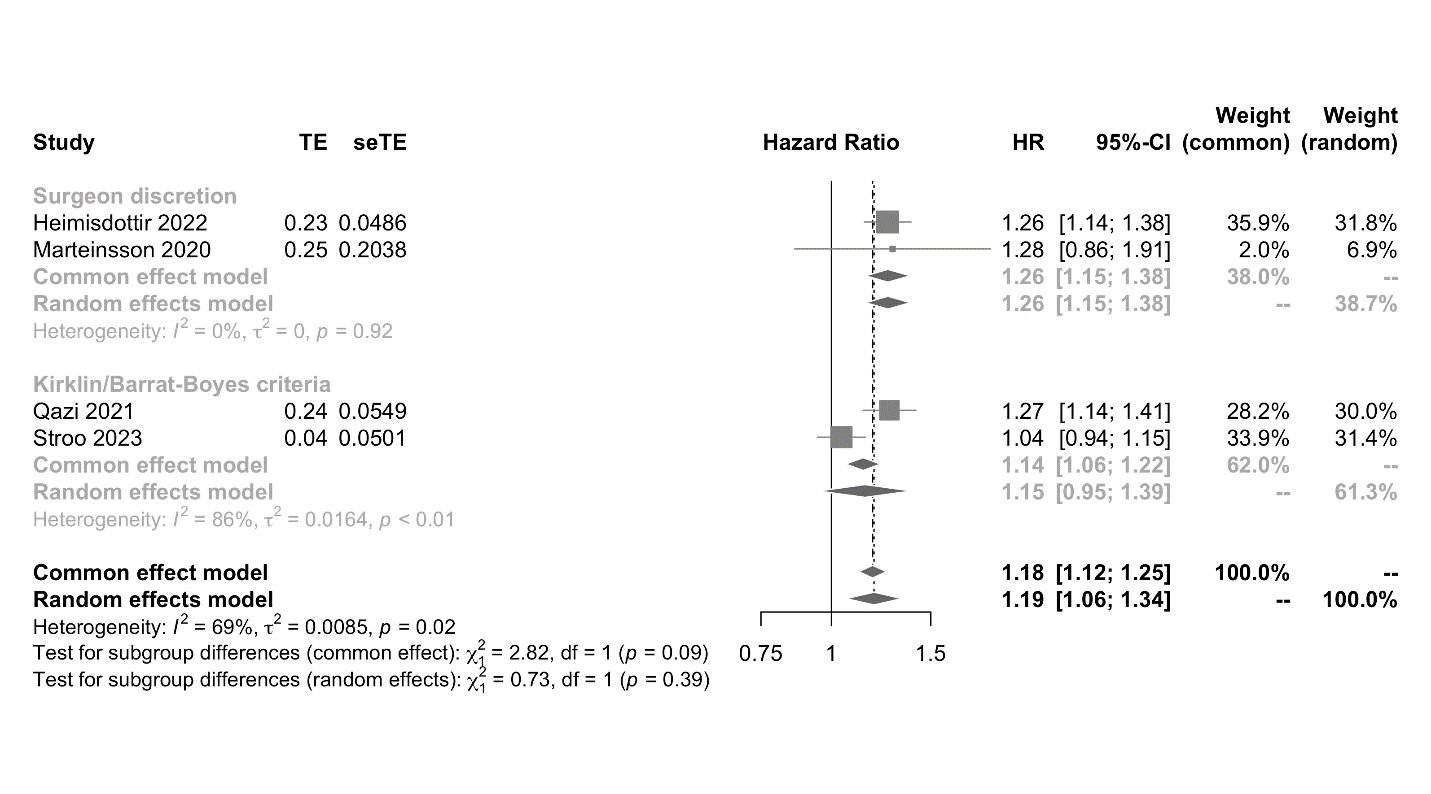


CI=confidence interval, HR=hazard ratio, TE= estimate of treatment effect, seTE=standard error of treatment estimate.

**Supplementary Figure 11**. Subgroup analysis of primary outcome based on single versus multi-centers studies.


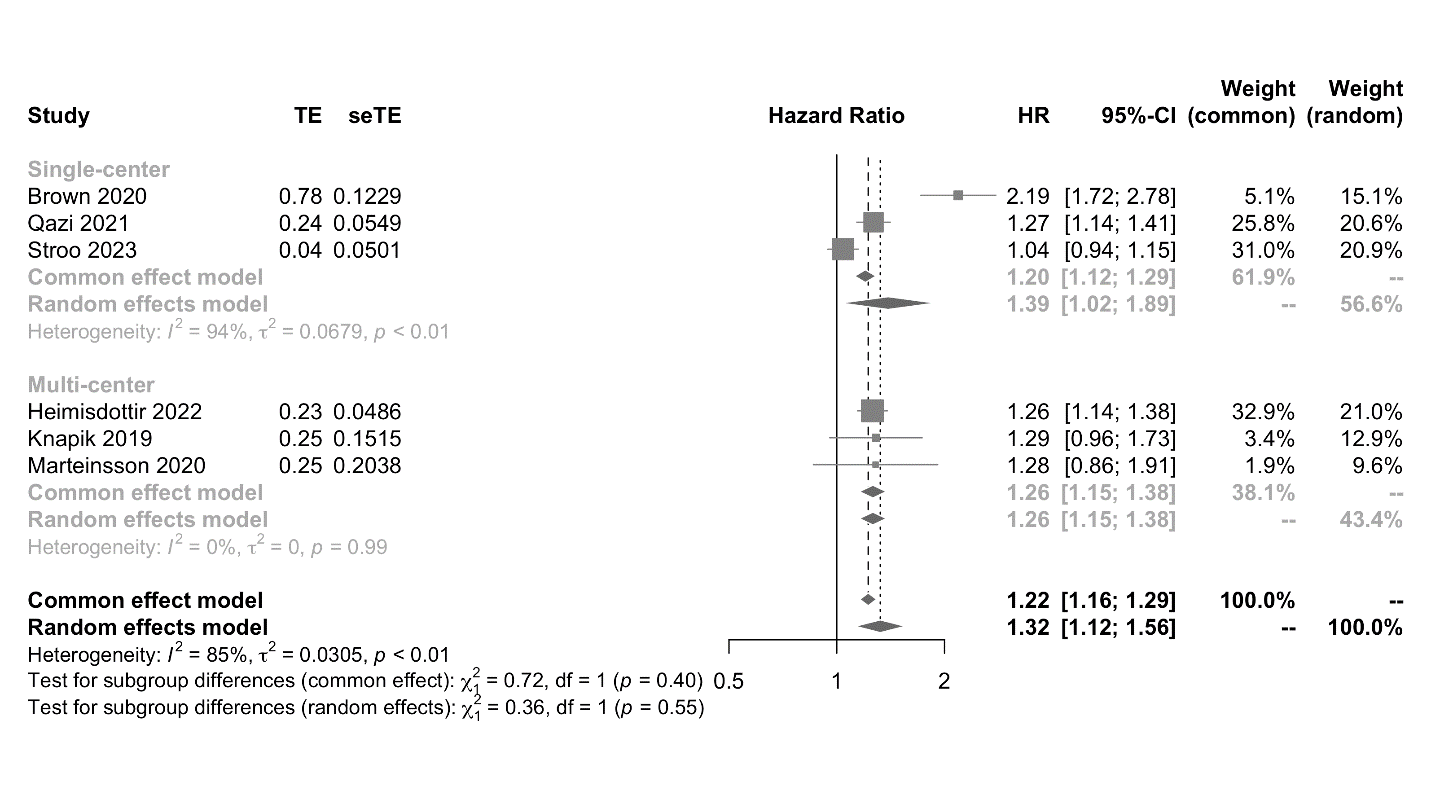


CI=confidence interval, HR=hazard ratio, TE=estimate of treatment effect, seTE=standard error of treatment estimate.
